# Bone tumors effective therapy through functionalized hydrogels: current developments and future expectations

**DOI:** 10.1080/10717544.2022.2075983

**Published:** 2022-05-25

**Authors:** Ruyi Shao, Yeben Wang, Laifeng Li, Yongqiang Dong, Jiayi Zhao, Wenqing Liang

**Affiliations:** a Department of Orthopedics, Zhuji People’s Hospital, Shaoxing, Zhejiang, China; b Department of Traumatic Orthopedics, Affiliated Jinan Third Hospital of Jining Medical University, Jinan, Shandong, China; c Department of Orthopaedics, Xinchang People’s Hospital, Shaoxing, Zhejiang, China; d Department of Orthopedics, Zhoushan Hospital of Traditional Chinese Medicine Affiliated to Zhejiang Chinese Medical University, Zhoushan, Zhejiang, China

**Keywords:** Functionalized hydrogels, bone tumors, targeted, therapy

## Abstract

Primary bone tumors especially, sarcomas affect adolescents the most because it originates from osteoblasts cells responsible for bone growth. Chemotherapy, surgery, and radiation therapy are the most often used clinical treatments. Regrettably, surgical resection frequently fails to entirely eradicate the tumor, which is the primary cause of metastasis and postoperative recurrence, leading to a high death rate. Additionally, bone tumors frequently penetrate significant regions of bone, rendering them incapable of self-repair, and impairing patients' quality of life. As a result, treating bone tumors and regenerating bone in the clinic is difficult. In recent decades, numerous sorts of alternative therapy approaches have been investigated due to a lack of approved treatments. Among the novel therapeutic approaches, hydrogel-based anticancer therapy has cleared the way for the development of new targeted techniques for treating bone cancer and bone regeneration. They include strategies such as co-delivery of several drug payloads, enhancing their biodistribution and transport capabilities, normalizing accumulation, and optimizing drug release profiles to decrease the limitations of current therapy. This review discusses current advances in functionalized hydrogels to develop a new technique for treating bone tumors by reducing postoperative tumor recurrence and promoting tissue repair.

## 1. Introduction

Bone cancer is a type of tumor that develops in the bone and kills normal bone tissues. It might be benign or cancerous. The tumor grows and compresses the normal bone tissues in both cases, however benign tumors lack the ability to metastasize and therefore do not spread to other organs of the body. Benign bone tumors can progress to malignancy and pose a risk if remain untreated. Benign bone tumors include osteochondroma, osteoma, osteoblastoma, fibrous dysplasia, and enchondroma (Hakim et al., 2015). According to the World Health Organization (WHO), bone cancers are classified as primary or secondary tumors (Sisu et al., 2012) and categorized over 45 distinct forms of bone tumors in 2002 based on their findings. Among the many kinds of bone tumors, Osteosarcoma is the most common and major type of bone tumor, accounting for 31.5% of all cases, followed by angiosarcoma (1.4%), malignant fibrous histitocytoma (5.7%), chondroma (8.4%), Ewing’s sarcoma (16%), and chondrosarcoma (25.8%) (Sisu et al., 2012; Jemal et al., 2005). Secondary bone tumors are usually malignant and develop as a result of soft tissue metastasizing tumors in the breast, liver, or lung. As per the American Cancer Society, the number of joint and bone cancer diagnoses and deaths rises each year (Miller et al., 2019).

Chemotherapy is the traditional postoperative treatment for bone tumors. Moreover, these drugs have the potential to cause systemic adverse effects such as liver failure, bone marrow suppression, and cardiac toxicity. Bone tumor therapy merges the complicated problems of tumor therapy with bone regeneration, which requires treatment with functionalized biomaterials. By developing novel alternate tumor therapy strategies based on biomaterials, these undesirable effects can be avoided by targeted delivery (Darge et al., 2019; Fan et al., 2019; Liao et al., 2020). But it is difficult to develop innovative techniques capable of preventing tumor recurrence while also promoting bone formation, requiring a multidisciplinary background to the research (Liu et al., 2019; Xue et al., 2020). However, researchers throughout the world have focused their efforts on finding solutions to these therapy’s issues for bone tumors and developing new therapeutic techniques which possess a great promise for developing treatments for bone malignancies, even though they are still in the initial phases of development.

Hydrogels are water-swellable polymeric materials having a three-dimensional (3D) network structure synthesized by crosslinking hydrophilic polymers. Hydrogels have a porous structure similar to that of the extracellular matrix (ECM), are biocompatible, and capable of loading growth factors, which results in effective bone defect repair. As such, they can be utilized as carrier materials for cells proliferation or bone growth to enhance the growth factors in bone tissue. Additionally, because its soft texture is similar to that of many soft biological tissues, it can help to lower the inflammatory reactions of nearby cells and tissues (Buwalda et al., 2017; Liu et al., 2019). Numerous researches have demonstrated the potential for hydrogels to be used in bone tissue regeneration. To be effective in treating bone cancers, the hydrogel should also have the potency to overcome tumor proliferation. It is strongly recommended that drugs or components be injected into the resected tumor region (Wu et al., 2018; Yang et al., 2020). Hydrogels may be used to deliver drugs in a sustained manner for tumor illumination (Ali Gumustas et al., 2016). Thus, hydrogels have established themselves as fascinating materials due to their remarkable biological activities and features. Numerous materials, including natural, synthetic, and hybrid polymers, have all been extensively used as the primary component of hydrogel for bone tumor therapy and tissue engineering (Culebras et al., 2021; Ma et al., 2021; Xu et al., 2021). Collagen, chitosan, hyaluronic acid (HA), gelatin, and sodium alginate are natural hydrogels that exhibit a high degree of biocompatibility, making them appealing biomedical materials for bone repair. Hydrogels derived from synthetic polymers including polyoxyethylene, polylactic acid (PLA), polyethylene glycol (PEG), poly-vinyl alcohol (PVA), and poly-caprolactone have a variable microstructure, a long shelf life, and high mechanical strength but lack biological activity. As a result, hydrogels are excellent options for bone restoration and cancer therapy. Certain hydrogels combine internal antitumor action and localized delivery in a single mechanism (Zhao et al., 2020). Hydrogels can be used to treat localized cancers without the need for oral or intravenous chemotherapy (Chen et al., 2019; Fu et al., 2019). The advancement of multifunctional hydrogels has expanded their uses beyond tissue repair to include bone repair and tumor cure. The perfect hydrogel scaffold must meet certain criteria, including biocompatibility, injectability, adherence to the cavities, high mechanical qualities, and porous structure (Yu & Ding, 2008). Among these, an injectable hydrogel can be used to fill or match irregular flaws using a noninvasive moderate gelation technique (Cui et al., 2020; Wasupalli & Verma, 2020).

This review describes various classes of bone cancers, the design of hydrogels; the evolution of hydrogels technology, and current advances in the use of functionalized hydrogels for bone tumor therapy. The main material discusses novel ways for the formation and treatment of functionalized hydrogels. The treatment of bone tumors with functionalized hydrogels is a critical area of research for bone tissue engineering. Additionally, functionalized hydrogels will be critical in the treatment of complicated diseases that incorporate both tumor therapy and tissue engineering (such as adipose tissue, bone and skin tissue engineering, etc.).

## 2. Bone cancer types

Bone cancers can broadly be divided into primary bone cancers (sarcomas) and secondary bone cancers (metastatic tumors of the bones). The predominant primary bone cancers which constitute nearly two-thirds of cancers include chondrosarcomas, osteosarcomas, and Ewing sarcomas. Primary bone cancers originate from primitive mesenchymal cells in the bones accounting for nearly 0.2% of all malignancies around the world. Secondary bone cancers occur due to various other advanced cancers in the body which spreads to the bones (Fan et al., 2019). The following section describes various types of these cancers to have a deeper understanding of the differences among them (Bădilă et al., 2021).

### 2.1. Bone sarcomas

Sarcomas are primary bone tumors originating from rich cell populations due to close interaction between cancer cells and local microenvironments’ cell types, e.g. mesenchymal stem cells, osteoblasts, cancer-associated fibroblasts, osteocytes, chondrocytes, osteoclasts, or immune infiltrates (Menéndez et al., 2021; Tzanakakis et al., 2021). The most common primary bone cancers are Ewing sarcomas, chondrosarcomas, and osteosarcomas constituting 16, 25, and 35% respectively of malignant primary bone tumors (Cortini et al., 2019; Menéndez et al., 2021). These tumors are rarely occurring and comprise overall <0.2% of all diagnosed cancers with an approximate incidence rate of around 0.9 per 100,000 individuals annually for all joint and bone malignancies (Franchi, 2012; Menéndez et al., 2021). Although they less frequently occur, they are challenging have high mortality rates, and pose an overall burden on healthcare sectors (Cortini et al., 2019; Thanindratarn et al., 2019). Sarcomas consist of diverse cells populations, including cancer stem cells (CSCs). CSCs have certain features of normal stem cells e.g. differentiation capacity and self-renewal. These CSCs can more precisely be termed ‘tumor-initiating cells’ because they can generate nearly all cell types usually present in a tumor (Steinbichler et al., 2018). Thus, CSCs produce a broad range of markers depending on the tissue of origin and cancer type (Fujiwara & Ozaki, 2016; Steinbichler et al., 2018).

#### 2.1.1. Osteosarcoma

The most frequently occurring primary bone tumor in young adults and adolescents is the osteosarcoma (Fujiwara & Ozaki, 2016; Tzanakakis et al., 2021). It has been reported that osteosarcoma predominantly occurs during the adolescent growth spurt, especially in the second decade of life with peak incidence cases at 16 and 18 years of life for girls and boys respectively (Taran et al., 2017). Osteosarcoma is regarded as aggressive cancer with a natural metastatic tendency where malignant cells lead to the formation of pathological bones (Gambera et al., 2021). Disorganized bone structures formation is the characteristic of this cancer presented with irregular clumps of osteoid or fine lacey trabecular pattern markedly different from the normal bone formation. The most commonly affected bones are the long rapidly growing ones including that of the juxta-epiphyseal regions (Rajani & Gibbs, 2012). No significant improvements in the survival rate of patients with osteosarcoma has been seen over the last few decades regardless of the use of multimodal and aggressive treatments with around 68% median five-year survival rate (Bădilă et al., 2021; Siegel et al., 2021). Chemo-resistance and prevention of metastasis are the most important challenges that impede the successful therapy of osteosarcoma (Miwa et al., 2021; Tzanakakis et al., 2021).

#### 2.1.2. Ewing sarcoma

Ewing sarcoma usually affects individuals in the initial 30 years of age and is characterized by round, small blue cells malignancy (Rajani & Gibbs, 2012). It is considered the second most frequently occurring malignant bone cancer in adolescents and children, with peak incidence at 15 years (Rajani & Gibbs, 2012; Thanindratarn et al., 2019; Tzanakakis et al., 2021). Adjuvant chemotherapy, local treatments (surgical or radiotherapy interventions), and neo-adjuvant chemotherapy are the conventional treatment approaches for such tumors. Surgical resection in healthy margins is preferred over radiotherapy alone (Thévenin-Lemoine et al., 2018). For localized Ewing sarcoma, the overall five-year survival rate is 70 to 80%, however, worse patient outcomes are observed in cases where large tumors, pelvic involvement, or incomplete tumor regression after chemotherapy is observed (Thanindratarn et al., 2019).

#### 2.1.3. Chondrosarcomas

Chondrosarcomas are mesenchymal cells tumors constituting nearly 10 to 20% of all malignant bone cancers. These tumors are characterized by distinctly differentiated cells with cartilage-like presentations (Rajani & Gibbs, 2012; Boehme et al., 2018). In contrast to Erving sarcoma and osteosarcoma, this cancer is frequently diagnosed in adults over 40 years of age having more than 70% of confirmed cases in this age group (Zając et al., 2021). Nevertheless, these are a group of heterogeneous tumors, both in terms of morphology and clinical presentations where nearly 10% constitute highly aggressive de-differentiated chondrosarcomas while the remaining 80–90% are conventional chondrosarcomas. Conventional chondrosarcomas frequently affect the femur, pelvis, ribs, humerus, and ilium. However, de-differentiated chondrosarcomas also affect the humerus, femur, and pelvis usually affecting non-chondroid and chondroid segments of these bones with osteoblastic or fibroblastic tissue presentation, indicative of two mesenchymal cell types differentiation in one tumor (Boehme et al., 2018; Zając et al., 2021). In addition, chondrosarcomas have usually a high tendency for metastasis to distant organs and usually metastasize to lungs (Tzeng et al., 2021). These tumors are diagnosed later in the disease stage due to the formation of anatomically deep lesions and combined with high resistance to conventional treatments (radiotherapy and chemotherapy) and even to targeted therapies resulting in poor prognoses (Rajani & Gibbs, 2012; Boehme et al., 2018; Miwa et al., 2021). Thus, the treatment options for chondrosarcoma are very limited where surgical resection is considered the only choice for such tumors in most cases (Miwa et al., 2021; Zając et al., 2021).

### 2.2. Bone metastases

The treatment of cancer becomes extremely difficult especially when metastasis occurs and it remains the most serious threat to human beings (Zhang et al., 2020). Bone is among the third most common sites in cancer patients and indicates a short prognosis with a mortality rate. It can occur in every type of cancer, however, is most commonly seen in prostate, breast, and lung cancer patients. Bone metastases are very painful and often cause significant morbidity represented by a wide range of skeletal events associated with considerable use of health resources (Svensson et al., 2017). The high morbidity and mortality of bone metastasis are because once tumor cells invade the bone and make a firm home in the skeleton, it can then rarely be treated successfully (Bădilă et al., 2021). Nevertheless, treatment is yet considered for pain alleviation and slowing metastasis development in such patients (Macedo et al., 2017; Ferracini et al., 2018; Cortini et al., 2019). Metastases and sarcoma represent similar niches and tissue microenvironments (Cortini et al., 2019). Growing evidences suggest that tumor cells remain dormant for decades in metastatic niche prior to their proliferation and metastasis development. Bone metastases cancer cells cannot directly destroy bones; however, they cause osteoclasts stimulation which leads to degradation of the bone extracellular matrix.

## 3. Conventional bone tumor therapy

Surgery, chemotherapy, and radiotherapy are the major treatments options for common malignant bone tumors. The morbidities due to bone tumors have faced a slow-down due to current treatments including chemotherapy, radiation therapy, and surgical treatments. Yet, these conventional treatment modalities owe a plethora of shortcomings (e.g. severe side effects, tumor recurrence, multi-drug resistance, large bone defects induction), thus limiting their efficacy (Bădilă et al., 2021). Tumor surgery's purpose is to completely resect the disease via substantial tumor excision, and cure requires total surgical resection. Amputation and limb salvage are two subcategories of surgical treatments. Even after surgical amputation, less than 20% of patients with high-grade conventional osteosarcoma survived without chemotherapy, showing the presence of preoperative micrometastases (typically pulmonary). If the final pathology shows that the tumor is low grade, excision is usually all that is necessary to treat it, and chemotherapy is avoided in such a case. Radiation therapy is a contentious topic in osteosarcoma treatment due to its unknown efficacy and risk of infection (Misaghi et al., 2018). Although osteosarcoma is a radioresistant disease, it can be treated with radiation to treat unresectable tumors after intralesional resection or symptomatic metastases (Luetke et al., 2014). Certain chemotherapy agents (e.g. ifosfamide (IFS), cisplatin, and a high dosage of methotrexate (MTX)) tend to enhance the effectiveness of local radiotherapy significantly. Osteosarcoma is considered to be radiation-resistant. Cryotherapy, thermal and radiofrequency ablation are the therapeutic methods under clinical stages, as well as chemo or angioembolization, which are further therapeutic techniques that are now in the experimental stage (Luetke et al., 2014; Lindsey et al., 2017).

Chemotherapy is one of the most important treatment options for osteosarcoma. Before the 1970s, osteosarcoma was not treated with chemotherapy, and mortality rates were extremely low. MD Anderson reported a 1972 study demonstrating a 50% mortality rate for osteosarcoma patients treated with chemotherapy over two years. In 1981, a prospective experiment was initiated to compare the outcomes of 32 patients treated with adriamycin (ADM), MTX (high dose), or bleomycin, actinomycin-D combinations, and Cytoxan to 27 patients treated without adjuvant chemotherapy (Misaghi et al., 2018). Various chemotherapy techniques have been investigated during the previous three decades and the procedure reported in the 1970s and early 1980s markedly increased survival rates of patients (Chou et al., 2008; Jaffe, 2009). A research group at Memorial Sloan Kettering Cancer Center established neo-adjuvant treatment and published numerous sequential series with progressively advanced chemotherapy regimens, such as that of the T-10 protocol (Meyers et al., 1992). The latter was carried out by multi-institutional organizations in the USA and Europe, which carried out confirmatory tests Simultaneously, several investigations were carried out employing alternative multi-valent regimens (Ferguson & Goorin, 2001). All of these efforts ultimately resulted in the development of the multi-agent neo-adjuvant strategy in combination with adjuvant chemotherapy.

The radiotherapy for bone cancer could cause bone death or osteoradionecrosis. In addition, radiotherapy can also induce new cancer in the exposed area (Hosoya et al., 2008). Chemotherapy is usually the traditional treatment for post-surgical bone cancer treatment. However, chemotherapeutic drugs are associated with severe systemic adverse effects including cardiotoxicity, liver damage, and suppression of bone marrow. The development of novel nanomaterials-based alternative or supplementary tumor treatment strategies can bypass these adverse effects by targeted delivery (Liao et al., 2021). Additionally, the traditional anticancer drugs are washed out quickly from blood being attacked by the immune system and macrophages. Thus, these agents remain for a very short time in the circulation and could not sufficiently confront cancerous cells rendering the chemotherapy nearly ineffective. Moreover, the majority of the conventional anticancer drugs are poor aqueous soluble making them unable to traverse bio-membranes and are thus poor bioavailable (Mousa & Bharali, 2011). The overexpression of multi-drug resistance proteins on tumors (e.g. P-glycoprotein) is another problem encountered in the treatment of cancers because these proteins work as efflux pumps, preventing drug accumulation inside tumors and leading to decreased therapeutic output (Brown & Links, 1999; Krishna & Mayer, 2000; Davis et al., 2010). Nanotechnology-based treatments are now being adopted to help address these issues associated with anticancer drugs and make them able to pass through biological barriers and successfully mediate molecular interactions. Nanomaterials have excellent desirable feature including large surface area and modifiable properties compared to conventional materials. Such materials include hydrogels, polymeric micelles, liposomes, dendrimers, nanotubes, and so on. Many of these nanotechnology-based products are already marketed and some are under research and evaluation for successful translation into clinics (Park, 2007; Praetorius & Mandal, 2007). For instance, calcium carbonate (CaCO_3_)- crosslinked hyaluronate (HA) nanoparticle was prepared via a ‘green’ process with responsive behavior toward tumor microenvironment to effectively deliver doxorubicin for the treatment of various stages of osteosarcoma. The developed system exhibited the triggered release at target site with improved survival time, and reduced adverse effects (Zhang et al., 2018). Reduction -responsive polypeptide micelles based on methoxy poly(ethylene glycol) -block-poly (S-tert-butylmercapto-L-cysteine) copolymers (mPEG113-b-PBMLC 4, P4M, and mPEG113- b-PBMLC 9, P9M) were developed to control the delivery of doxorubicin in OS therapy (Yin et al., 2020). Curcumin (CUR) and cisplatin (CDDP) are co-incorporating in CaCO_3_ nanoparticles using an easy one-pot strategy in sealed containers and in situ synthesized polydopamine (PDA) as a template for enhancing mitochondrial dysfunction caused by Ca^2+^ overload in cancer therapy while minimizing unwanted off-target consequences (Zheng et al., 2021).

## 4. Current hydrogel-based strategies for the bone tumors therapy

**Figure 1.**
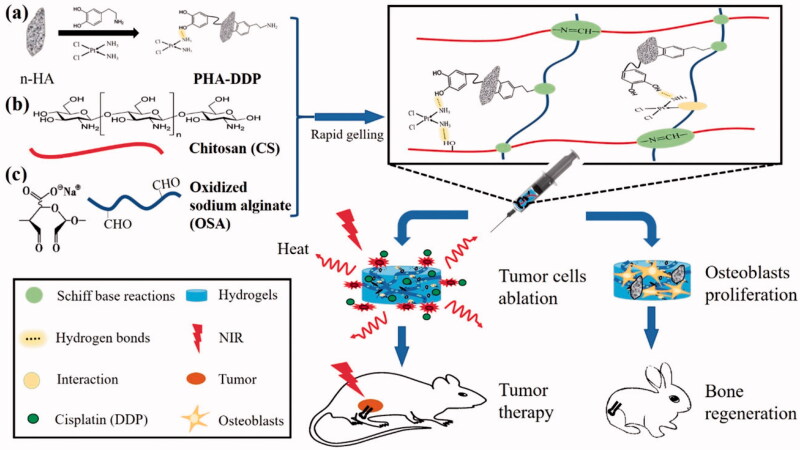
The synthesis of bifunctional OSA-CS-PHA-DDP hydrogels and their bio-applications are depicted schematically. Reproduce with permission from reference (Luo et al., 2019).

The high proliferative and penetrating ability of cancer cells (osteosarcoma) continues to be the main cause of the long-term survival rate of patients associated with osteosarcoma. As a result, new therapeutic options for osteosarcoma are critically needed. The development and applications of nanomaterials in bone ailments have already displayed promising potential. Biomaterials used in bone tumor therapy must accomplish the tasks such as they must target and killing tumor cells as well as stimulating bone repair. Among the various scaffolds used for bone tumors, hydrogels show more benefits over the other counterpart nanosystems and thus are considered the recent research hotspot. These materials show excellent biocompatibility, good drug-loading capacity, biodegradability, and programmable drug release potential and more importantly show less toxicity than other carrier systems (Chindamo et al., 2020). Advances in technology have made hydrogel’s production and application more efficient over the past few decades, and one of the most important branches has been used in biological tissue regeneration. These advancements open up new avenues for overcoming the disadvantages of conventional bone repair materials, such as their poor mechanical characteristics and limited bioactivity, through rational design and manufacture of hydrogels with desirable topologies and characteristics. Recently, hybrid and multifunctional or bi-functional hydrogels have been developed to simultaneously enhance new tissue formation post-surgical resection of the tumor as well as prevent its recurrence (Liao et al., 2021; Chen et al., 2022). Multifunctional hydrogels for bone regeneration have also been reported recently which showed better and faster bone repair results in vivo in the rabbit model (Liu et al., 2020). Luo et al reported the synthesis of polydopamine and cisplatin decorating an n-HA surface loaded in chitosan/alginate hydrogels for photothermal therapy and chemotherapy for 4T1 breast tumor-bearing mice (Luo et al., 2019) (Figure 1).These reports indicate the excellent potential of hydrogels for effective bone tumor therapy and regeneration.

### 4.1. Design strategies of hydrogels for bone diseases and tissue engineering

**Figure 2.**
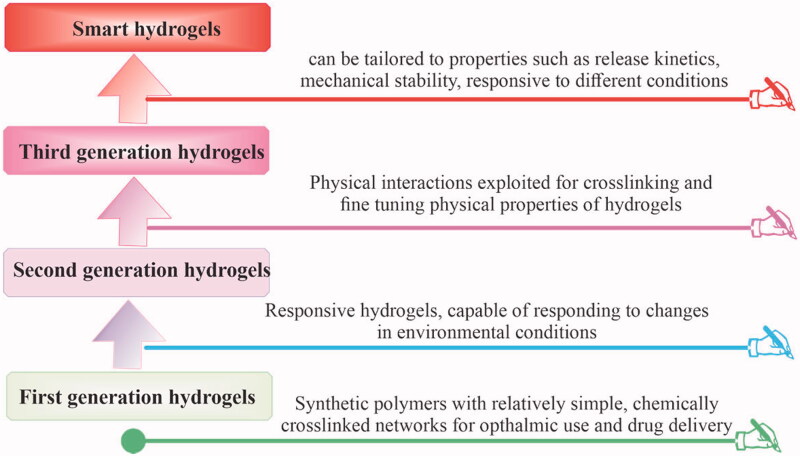
Developmental stages and evolution of hydrogels.

The hydrogels’ development has seen four developmental stages as shown in Figure 2. For the first time in 1960, Wichterle and Lim reported the porous synthetic polymer of poly-2-hydroxyethylmethacrylate (PHEMA) to retain high water content and adjustable mechanical characteristics which they successfully used in contact lenses (Wichterle & Lim, 1960; Buwalda et al., 2014). Subsequently, research on hydrogels grown and shifted from simple single polymer networks e.g. poly(vinyl alcohol) (PVA), poly(ethylene glycol) (PEG), and PHEMA to stimulus-responsive and in situ second-generation hydrogels (Hodgson et al., 2017). The pluronic hydrogel was prepared by Nalbandian et al. (1972) which could be employed as artificial skin in treating thermal burns and used for the controlled release of silver lactate or silver nitrate antimicrobials. In the third-generation hydrogels, physical interactions e.g. inclusion complexation and stereo-complexation were utilized as crosslinking tools in combination with hydrophobic interactions to improve and fine-tune the release characteristics (targeted release) of hydrogels (Buwalda et al., 2017). An oligo(lactic acid) side chain stereo-complex hydrogel was prepared by van Nostrum et al. (2004) with phosphonanted hydrolytic polymaleic anhydride which showed a considerably longer degradation time compared with dextran stereo-ployocomplex hydrogels. At present, advanced chemical techniques of polymer and organic chemistry and nanotechnology are applied in hydrogels’ research which yields the development of ‘fourth-generation hydrogels’ with exceptional structural and new functional features, suggesting their use for more accurate and targeted delivery to bone and other tissues (Buwalda et al., 2014).

### 4.2. Components and sources of hydrogel materials

**Figure 3.**
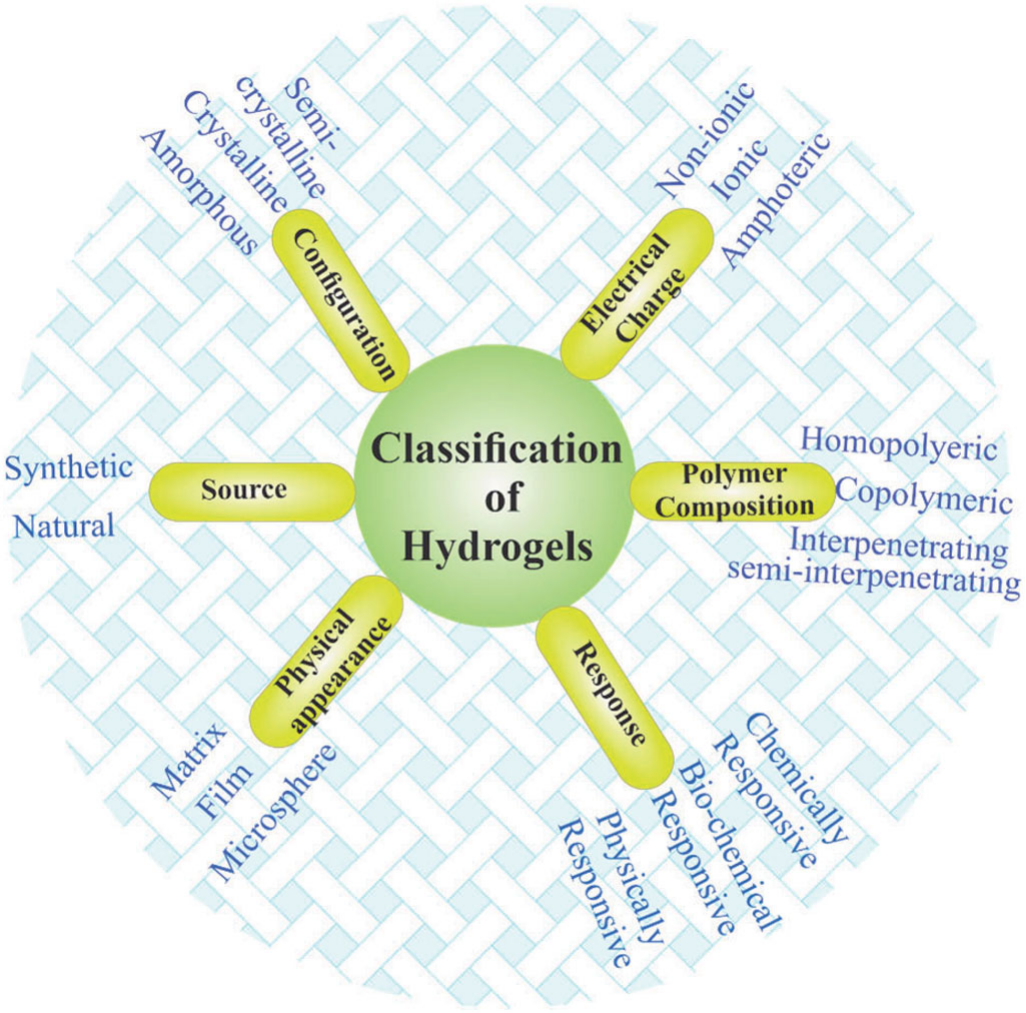
Classification of hydrogels based on various features.

Hydrogels can broadly be categorized into various types depending on their different properties including polymeric composition, sources, crosslinking types, degradation rates, physical properties, network charge, and responsiveness to environmental conditions as shown in Figure 3. Hydrogels can be prepared from natural materials obtained from natural sources and synthetic materials synthesized via the use of synthetic chemistry and chemical reactions (Ahmed, 2015). Most natural polymers are aqueous soluble and hydrogels made from natural source polymers generally show good biodegradability and biocompatibility (He et al., 2018). The lipophobic surfaces of natural polymers allow easy cells adherence, proliferation, and differentiation, however, the stability and mechanical properties of such materials are poor (Huang & Yang, 2008). Natural polymers commonly used for hydrogels synthesis include Gelatin (Chen et al., 2004), alginate (Kolambkar et al., 2011), hyaluronic acid (Li et al., 2015), chitosan, and dextran (Cascone et al., 1999), to mention a few. A gelatin hydrogel was prepared by Sasaki et al. (2013) containing basic fibroblast growth factor (bFGF) and demonstrated that the hydrogel was safe for the injured site and it can promote proximal sesamoid fractures healing. They also suggested that by controlling the degree of hydrogels crosslinking, its degradation can be controlled.

Synthetic polymer-based hydrogels can control synthesis and advantages of reproducibility (Amini & Nair, 2012). Nevertheless, the material safety and biocompatibility of synthetic polymers are the main issues. In addition, synthetic polymers possess inferior biological activities in comparison with natural biomaterials (Qing et al., 2013). Polyethylene glycol (PEG), poly-caprolactone (PCL), poly (N-isopropylacrylamide) (PNIPAAm), poly(L-glutamic acid) (PGA), polypropylene fiber (PPF), and poly-vinyl alcohol (PVA) are the examples of most common synthetic polymers used for the production of hydrogels (Yue et al., 2020). PEG is a promising polymer able to bind to lipophilic biodegradable polymers e.g. PCL and poly(lactic acid) (PLA) and create amphiphilic polymer biomaterials for bone and tissue engineering (Wang et al., 2019). The synthetic materials can be combined in different proportions for the construction of desired features hydrogels system. A triblock copolymer of PEG-PCL-PEG (PECE) combined with collagen and nano-hydroxyapatite (n-HA) based thermosensitive injectable hydrogel system was prepared by Fu et al. (2012) which combines the advantageous features of PECE hydrogels, collagen, and n-HA fillers and demonstrated excellent bone regeneration property in comparison with natural repairing process.

Hydrogels can be divided into homo-polymeric, co-polymeric, interpenetrating polymeric network (IPN), and semi-interpenetrating polymeric network (semi-IPN) hydrogels on the basis of the crosslinking mechanism (Ullah et al., 2015). In homo-polymer hydrogels, the crosslinking network is made by the polymerization of a single water-soluble monomer (Iizawa et al., 2007), whereas, in co-polymeric hydrogels, it is formed by two or more different monomer units, having at least one hydrophilic monomer unit (Lipatov, 2002). When one linear polymer penetrates another crosslinked network without any chemical bonds, the polymer system is termed semi-IPN (Zhang et al., 2009). It is mostly synthesized through the use of a polymerization initiator in a monomer solution to bind two polymers together and immersed in pre-polymerized hydrogels (Lipatov, 2002). Semi-IPN can efficiently show a dynamic response to temperature or pH due to its un-restricted elastic network and is considered promising for bone and tissue engineering. Inter-penetrating network hydrogels can generate dense hydrogel matrices with effective drug-loading, higher mechanical features, and show controlled physical characteristics (Maolin et al., 2000; Lipatov, 2002; Liou et al., 2010).

Certain hydrogels have the capability to respond to external stimuli and such hydrogels are termed stimuli-responsive hydrogels. The network structure, volume, mechanical properties, and other features of such hydrogels change when the stimulus is applied. These hydrogels can be physically responsive, chemical responsive, and biochemical responsive hydrogels based on their response to the type of external stimulus. Temperature, light, pressure, electric, and magnetic fields are examples of physical stimuli, while chemical agents, ionic strength, and pH constitute chemical stimuli. In addition, the hydrogels that respond to enzymes, ligands or antigens, and other biochemical drugs are termed biochemical stimuli-responsive (Ahmed, 2015). Thermo-responsive hydrogels are usually liquid at ∼ 25 °C temperature which is quickly gelled at physiological or specific local tissue temperature and are commonly used as injectable hydrogels for bone and tissue engineering (Nagahama et al., 2013; Sood et al., 2016). Such hydrogels have the ability to reach the defective bone site with the use of minimally invasive surgery, can be used for fixation of injured bone tissue, treating any shape of bone deformity, and might be utilized for delivery of drugs (Soundarya et al., 2018).

### 4.3. Synthesis and preparation of hydrogels

Hydrogel networks are primarily formed by two types of crosslinking: chemical and physical. Ionic, electrostatic, and hydrophobic interactions crystallization and hydrogen bonding all contribute to the connectivity of physical hydrogels (Akhtar et al., 2016). Chemically crosslinked hydrogels can be synthesized using a variety of processes, including Michael addition, Schiff base reactions, free radical polymerization, Diels-Alder cycloadditions, and other click chemistry reactions (Huang et al., 2017). Macromolecules fold into scaffolds with well-defined structures and functions owing to physical noncovalent bonding mechanisms (Zhang & Khademhosseini, 2017). Due to its ability to self-assemble under particular conditions and lack of dependency on crosslinkers, physical hydrogels are becoming increasingly popular among researchers. Recent reports revealed that a wide range of physical injectable hydrogels, such as stress-sensitive or ion-sensitive hydrogels, can now be made without any chemical discomfort (Ren et al., 2015). Ionic interactions are frequently employed to synthesize the hydrogels which were based on natural polysaccharides, such as calcium silicate/sodium alginate composite hydrogels. It enhances the adhesion, proliferation, and differentiation of osteoblasts and angiogenic cells and has a wide range of potential applications in bone regeneration and tissue engineering (Han et al., 2013).

In comparison to physical approaches, chemical methods significantly increase the control of the crosslinking process's flexibility and spatiotemporal accuracy, which results in a more stable hydrogel matrix (Zhang & Khademhosseini, 2017). The Michael addition process may be performed in aqueous environments, at physiological pH, and ∼ 25 °C, making it an attractive technique for the preparation of biomimetic hydrogels (Nair et al., 2014). However, the significant cytotoxicity of the high-efficiency reaction involving acrylate Michael acceptors can be mitigated to some extent by decreasing the reaction duration (Huang et al., 2017). It is possible to build cell-friendly materials using the Schiff base reaction between an aldehyde group and an amine under physiological conditions, which quickly produces a nontoxic gel with excellent biocompatibility (Lü et al., 2015). Schiff base reactions to generate self-healing hydrogels are also possible because of the dynamic equilibrium of Schiff base bonds (zur Nieden et al., 2015). For cell and growth factor encapsulation, click chemistry is being investigated and exploited to develop new, candidate materials with fascinating features (Diehl et al., 2016).

In spite of the promising potential of intelligent hydrogels in biomedical fields, they were made using traditional procedures and still face a number of major obstacles, such as uncontrolled stimulus-response and low responsive sensitivity. Recently, it was demonstrated that reversible addition-fragmentation chain transfer (RAFT) polymerization is an adaptable approach for fabricating intelligent hydrogels with improved stimulus-response features, attributed to its ability to effectively build hydrogel precursors with well-defined structures, including block copolymer, graft copolymer, and star copolymer (Xian et al., 2020).

### 4.4. Hydrogel scaffolds produced through 3D printing

**Figure 4.**
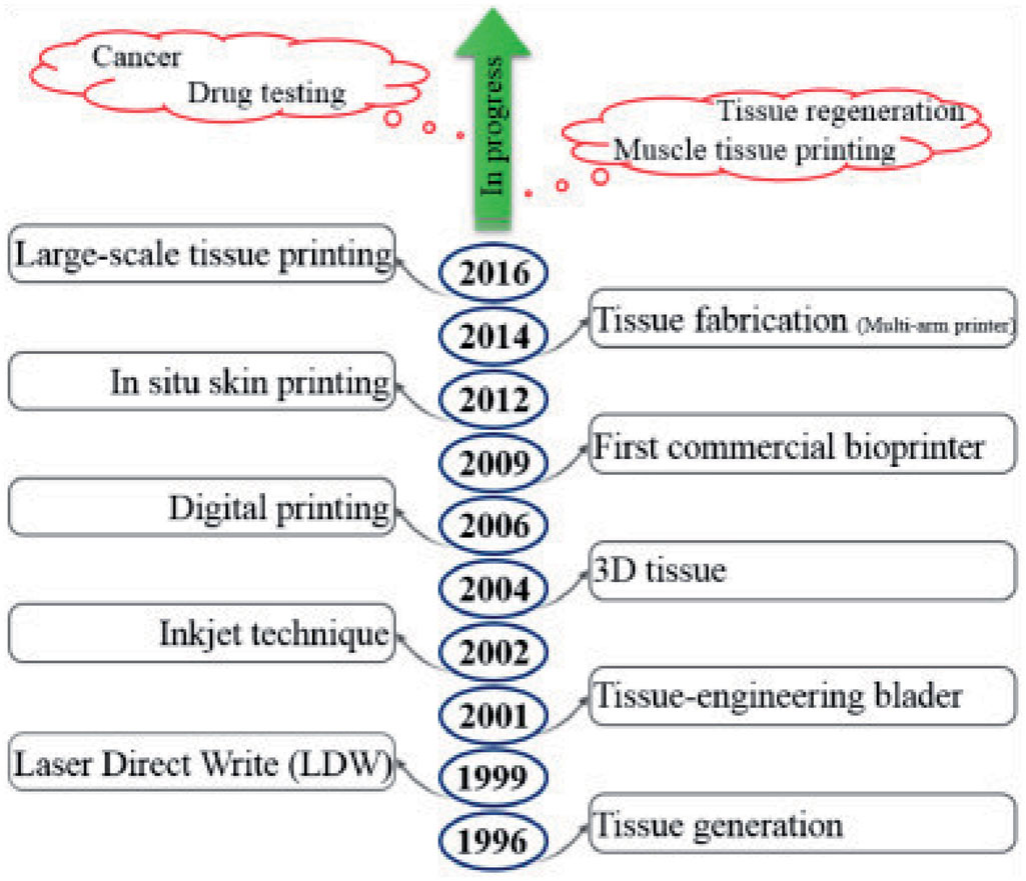
A schematic representing the progress of 3D bioprinting techniques (Vanaei et al., 2021).

Numerous strategies for fabricating hydrogel scaffolds for efficient treatment of major bone defects have been established, including skull defects, craniofacial bone fractures, and subchondral bone injury (Tan et al., 2021; Zhou et al., 2021). Previously, these bone regeneration materials were typically intended to be devoid of biological activity; however, scientists are currently working on producing bioactive hydrogel scaffolds with acceptable mechanical properties and biological properties to produce a more desirable healing effect (MM, 2008). Furthermore, 3D printing technology has attracted the attention of a range of interdisciplinary societies, including those concerned with bone and biomaterials research (Murphy & Atala, 2014). A schematic representing the progress of 3D bioprinting techniques are shown in Figure 4. Apart from bone tissue regeneration, it has been revealed that novel functional hydrogel frameworks are capable of bone regeneration tumor therapy simultaneously. As a result, such functional scaffolds can be employed to repair damaged tissue caused through surgery while also removing any remaining tumor cells, achieving the goal of bone tumor therapy.

In comparison to radio/chemotherapy, magneto/photothermal therapy has significantly lesser or minimum adverts effects and can successfully destroy tumor cells without causing damage to adjacent healthy tissue as cancerous cells have higher sensitivity to heat relative to healthy cells due to the differential expression of heat shock proteins (HSPs) involved in the cellular defense system against various stressors including heat (Di Mauro et al., 2018). Hence, the temperature elevation generated through functional scaffolds results in late progressive apoptosis, irreversible protein denaturation, and cell membrane damage. Thus, functional scaffolds with outstanding magneto/photothermal properties can be used locally as magneto/photothermal agents. Additionally, these functional hydrogel scaffolds are biocompatible, increase bone mesenchymal stem cell (MSC) migration, adhesion, differentiation, proliferation, and enhance new bone formation in vivo (Przekora, 2019). Ma et al. developed a biomaterial scaffold modified with graphene oxide (GO) that has improved photothermal characteristics (Ma et al., 2016). The unique photothermal impact of functional scaffolds can be controlled to efficiently ablate tumor cells and prevent tumor growth in mice. Elevated temperatures caused through functional scaffolds have been shown to greatly decrease tumor cell proliferation and significantly increase the apoptotic process of tumor cells. In addition, due to the specific protein absorption and bioactive groups of GO, functional scaffolds increased the osteogenic growth of rabbit bone MSCs relative to pure biomaterial scaffolds. Additional research suggests that functional scaffolds may considerably contribute in the treatment of bone tumors because they promote bone growth in the body more effectively than pure scaffolds (Li et al., 2021).

**Figure 5.**
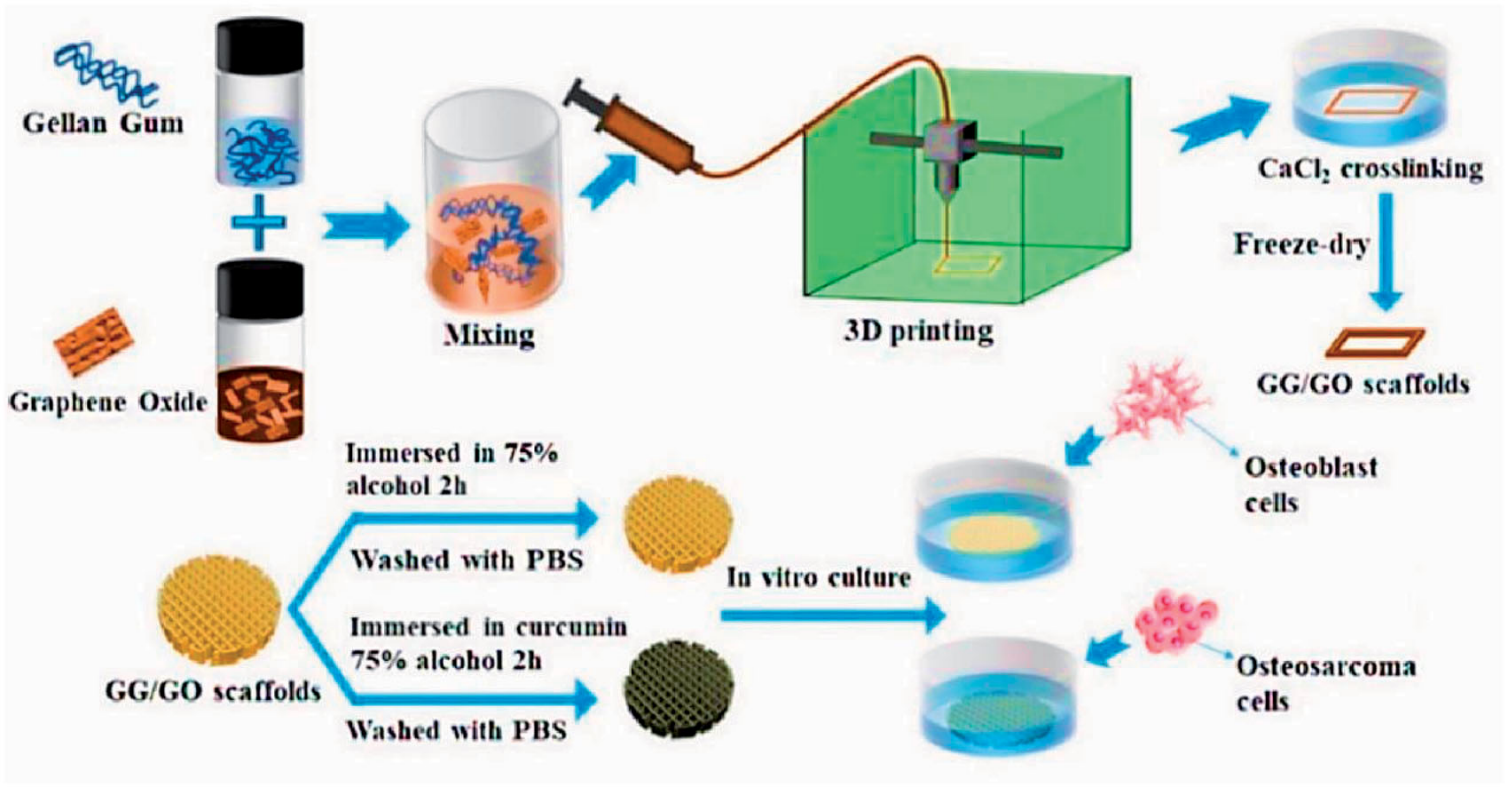
Scheme showing the fabrication process of GG/GO and GG/GO/Cur scaffolds. Reproduced with permission from reference (Zhu et al., 2021).

Hydrogel-based scaffolds with on-demand delivery of drugs have attracted considerable interest for application in targeted tissue engineering and cancer therapy. On the other hand, the majority of drug-loaded hydrogels are unsuitable for long-term drug delivery due to uncontrolled drug diffusion through swelling hydrogels. Poly-caprolactone (PCL) was used in the production of a core/shell fiber scaffold, which was 3D printed over alginate-gelatin hydrogel scaffolds, by Liu et al. (2021). Coated PCL may help to prevent drugs from diffusing freely from the core gels. Following that, the Gel/PCL core/shell scaffolds were coated with polydopamine (PDA), endowing them with significant photothermal properties. Thus, on-demand drug release initiated by a near-infrared (NIR) laser was obtained in this system as a result of the core gels' caused by the thermal sol-gel transition. The combination of photothermal therapy and the release of doxorubicin may be able to prevent or eliminate tumors in vivo and in vitro. Furthermore, the scaffold based on the Gel/PCL/PDA core/shell could be used to promote wound healing. As a result, they described the potential to be used in tissue regeneration and localized cancer therapy. The scaffolds could be implanted at the resection site of cancer patients who have had surgical resection to kill remaining or recurrent cancer cells and repair tissue defects caused by surgery. 3D printing technology outperforms conventional tissue engineering scaffold methodological approaches in getting higher structural complexity, patient-specific needs, and flexibility compared to the traditional approaches. Printing ink compounds that are both printable and biocompatible continues to be a significant problem. Zhu et al. developed a hydrogel bio-ink with outstanding thixotropy and recovery capabilities for extrusion 3D printing by mixing gellan gum (GG) with GO (Figure 5) (Zhu et al., 2021). The 3D-printed GG/GO scaffold is a porous and uniform structure, retaining a high degree of model fidelity. The bi-functional GG/GO/Cur scaffold substantially suppressed the growth of the human osteosarcoma cell line such as MG-63 and efficiently induced tumor cell death in vitro, as evidenced by cellular proliferation and adhesion to the printable 3D scaffold loaded with curcumin. At the same time, the scaffold could promote in the adhesion and growth of mouse osteoblast cells such as MC3T3. Thus, the bi-functional GG/GO/Cur scaffold is predicted to be employed to repair bone defects caused by surgery while simultaneously killing any remaining tumor cells, so attaining the dual goal of tumor therapy and bone restoration.

### 4.5. Injectable hydrogels

**Figure 6.**
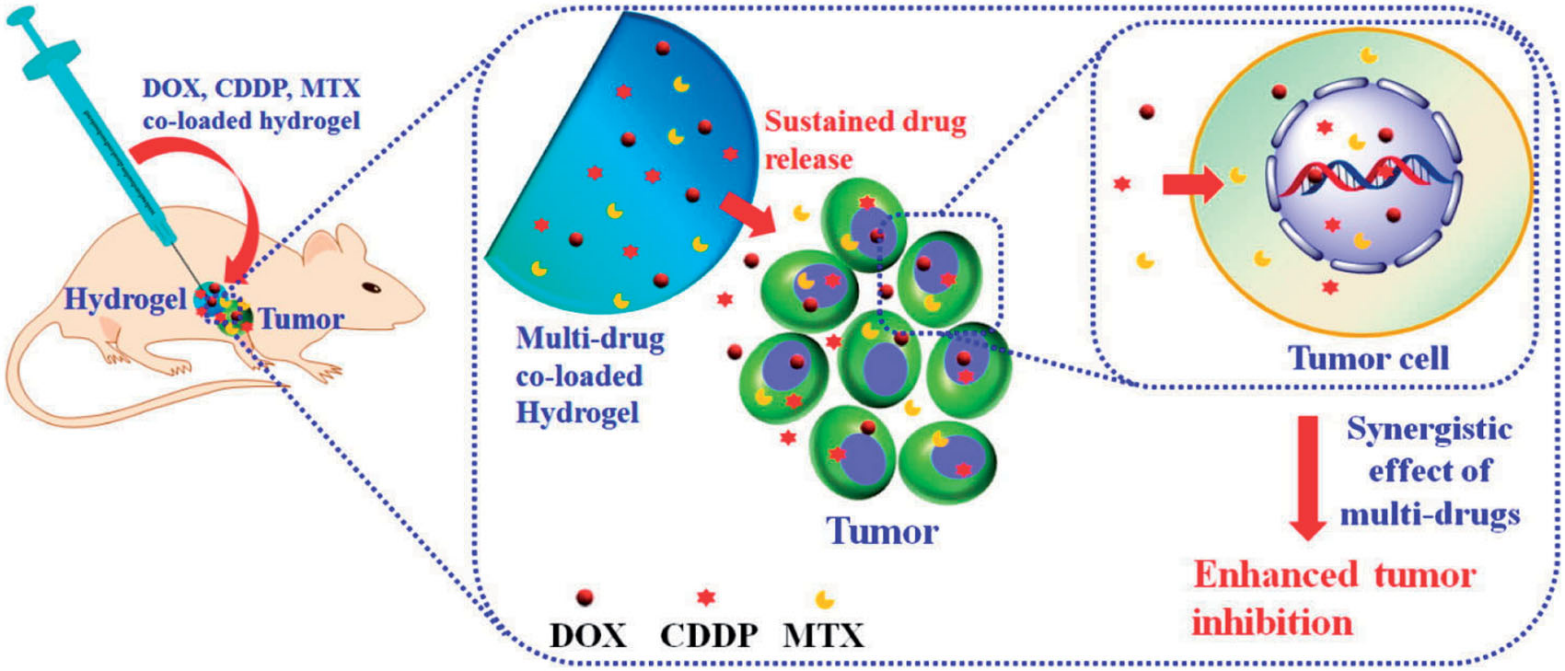
Schematic representation of the synergistic delivery of DOX, CDDP, and MTX through injectable hydrogels. Reproduced with permission from ACS 2015 (Ma et al., 2015).

Injectable biomaterials enable minimally invasive procedures and offer a wide range of uses in bone regeneration. In comparison to conventionally examined scaffolds, injectable hydrogels have several advantages, such as being unique and irreplaceable for filling irregular defects, improving patient compliance with bone injury, and allowing tissue regrowth and regeneration in situ (Hou et al., 2004). Ma, H., et al. synthesized the injectable hydrogels for the synergistic delivery of doxorubicin, cisplatin and methotraxate for osteocarcinoma treatment. Schematic for their preparation are provided in Figure 6. Several biomaterials, such as HA, chondroitin sulfate, chitosan, collagen, and polypeptide, can be used as appropriate substances for the production of injectable hydrogels. According to the results of biological evaluations in vivo, they all possess excellent biocompatibility and biodegradability.

### 4.6. Collagen-based hydrogels

**Figure 7.**
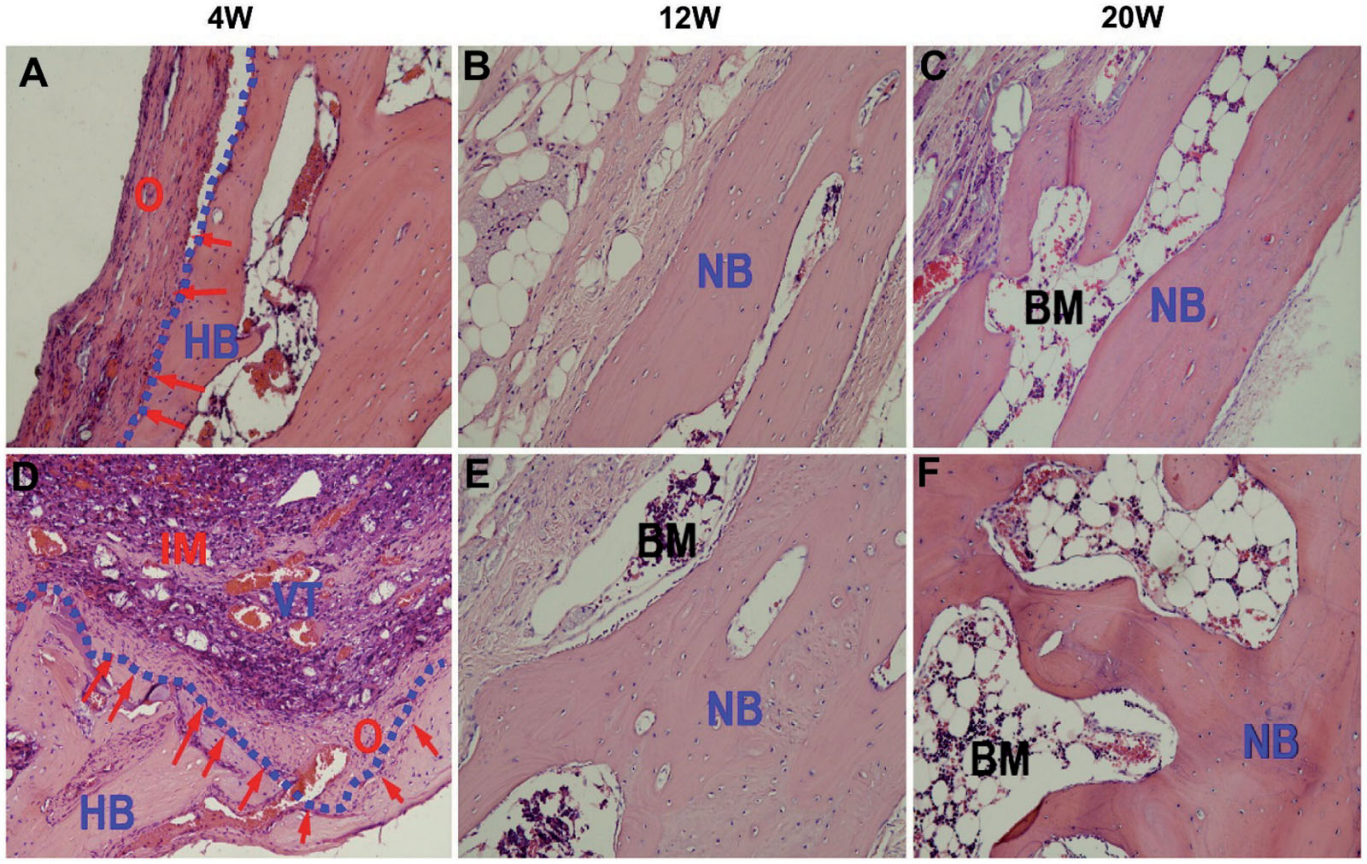
H&E staining of cranial bone defect sections in the control group (A–C) and in the PECE/Collagen/n-HA hydrogel composite treatment group (D–F). The treatment group had no significant foreign body reaction and inflammatory action. Two groups permitted bone ingrowth, but the treatment group demonstrated more rapid and successful osteogenesis at the defect site than that of the control group. The following abbreviations are used: BM: bone marrow; HB: host bone; IM: implanted material; NB: new bone; VT: vascular tissue; O: osteoid. The red (arrows) represent the new osteoid that formed at the HB edge. The blue (dotted line) denotes the junction of the HB with the defect site (Fu et al., 2012).

Collagen is a fibrous protein with unique physicochemical and structural properties that have been widely used in biological applications. Collagen-containing cells can move directly into hydrogel scaffolds through integrin, suggesting that collagen may be crucial for osteoconduction. Collagen, as a part of cartilage, is an excellent substance for regenerating bone tissue, particularly cartilage tissue, due to its ability to promote cell differentiation and intrinsic biocompatibility. Collagen's key disadvantages are its weak mechanical characteristics and rapid disintegration rate, which inhibit MSC differentiation and proliferation, resulting in an insufficient effect of bone repair (Huang et al., 2017). To address the aforementioned concerns, great effort has been made to improve the performance of injectable hydrogels based on collagen in recent research, which has demonstrated a favorable therapeutic impact in a bone deficiency model. Hydrogels made with a PEG-PCL-PEG copolymer (PECE), collagen, and n-HA, collagen-based composite hydrogel, were successfully developed by Fu et al. Because the pure collagen hydrogel group lacks thermal sensitivities, the researchers covalently crosslinked the PECE into the collagen chain, so introducing heat sensitivities to the composite hydrogel (Fu et al., 2012). To investigate the temperature-sensitive property of composite hydrogel, a test tube inversion approach and rheological analysis were used. The composite hydrogel exhibited freedom flow characteristics at room temperature but began gelating around 37 °C. Additionally, the ability of the composite hydrogel scaffolds to regenerate bone was assessed by injecting them into cranial lesions in New Zealand White rabbit models for 20 weeks. In comparison to the control group, the group injecting the hydrogel scaffolds revealed a greater capacity for bone repair, as determined by histological sections and micro-computed tomography images which are provided in Figure 7. Figure 7(A–C) depicts the variations in the left blank control, whereas Figure 7(D–F) depicts the formation of new bone in the right defects exposed to the PECE/Collagen/n-HA hydrogel composite for the same period as the left. There is a thin layer of new osteoid on the defect edges and the remaining bottom bone surface in both groups at 4 weeks. A high number of osteoblasts formed in the middle location of the defects (Figure 7(D)), where some osteoid and a lot of blood vessel tissue had formed on the new bone matrix However, in the blank defect, just a few new osteoid tissues were found (Figure 7(A)). However, we observe some inflammatory cells in the center of the treated defect, indicating that a modest inflammatory response may have been induced by the implant's degradation. By week 12, the newly generated osteoid had matured into the cortical bone and had fused with the surviving host bone. Nonetheless, as compared to the blank defect (Figure 7(B)), the treated defect's cortical bone layer was thicker, and some bone marrow was visible at the outside edge of the freshly created bone. After 20 weeks of surgery, a noticeable variation between the two groups persisted (*p* < .05), the blank defect (Figure 7(C)) was partially filled with newly formed cortical bone, but the treated defect (Figure 7(F)) was entirely padded by newly formed cortical bone tissue under the same magnification

**Figure 8.**
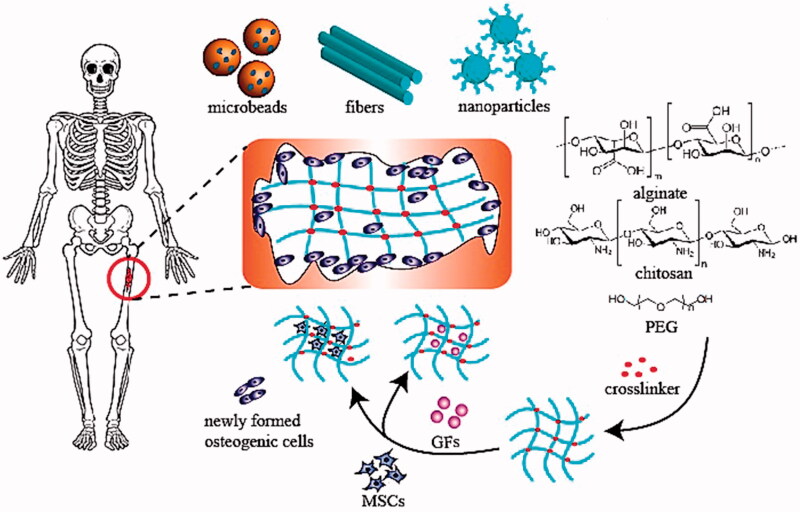
Schematic illustration of hydrogel-assisted bone regeneration (Bai et al., 2018).

As a consequence of the in vivo investigations, the PECE/collagen/n-HA composite hydrogel was found to be more biocompatible and capable of bone regeneration than the control group.

Fitzgerald et al. used collagen-based scaffolds to design a cell culture model in a 3D arrangement for prostate cancer bone metastasis to classify the model and analyze its potential for testing the delivery of gene therapies targeted at bone metastases (Fitzgerald et al., 2015). PC3 and LNCaP cell lines from prostate cancer were grown in two-dimensional (2D) conventional culture and particularly in comparison to 3D cell growth on three scaffolds based on collagen, including collagen and collagen composites containing nano-hydroxyapatite or glycosaminoglycan. Matrix metalloproteinase (MMP), viability, prostate-specific antigen (PSA), and cell proliferation production were all determined in the 3D model. In comparison to 3D, chemosensitivity to docetaxel therapy was assessed in 2D. Nanoparticles (NPs) with siRNA made with a functionalized cyclodextrin were used to evaluate gene silencing. On the scaffolds, PC3 and LNCaP were both actively invaded and proliferated. In PC3 cells, 3D culture resulted in decreased MMP1 and MMP9 secretion. In comparison, LNCaP cells grown in 3D released increased amounts of PSA, notably on the collagen and glycosaminoglycan scaffold. Both 3D-grown cell lines exhibited greater resistance to docetaxel therapy. In the 3D model, the cyclodextrin siRNA NPs were taken up by the cells and knocked off the endogenous GAPDH gene. To conclude, the development of a unique cell culture model in a 3D arrangement for prostate cancer bone metastasis has started, resulting in the first effective delivery of gene treatments in a 3D in vitro model. Further development of this model will aid in elucidating the pathophysiology of prostate cancer as well as accelerating the development of effective treatments that can penetrate the bone microenvironment for prostate cancer therapy.

### 4.7. Hyaluronic acid-based hydrogels

HA, β-1,4-d-glucuronic acid-β-1,3-N-acetyl-d-glucosamine, is a significant GAGs found in bone tissue, particularly cartilage, and may be the primary component of synovial fluid. In comparison to collagen, HA comes from a broader range of sources and is less expensive. Additionally, HA, as an endogen, has lower immunogenicity than collagen. Specifically, it has been demonstrated that HA has a role in anti-inflammatory effects, degradation, proteoglycan release inhibition, and prostaglandin production inhibition, and cellular signaling (Kim et al., 2011). Furthermore, HA is a macromolecule composed of linear polysaccharides with a molecular weight of 100–8000 kDa with a structural formula that contains free OH, N-acetyl, and COOH groups. All of these classes are primary targets for enhancing structural organization and physiochemical properties. Furthermore, HA can be destroyed in living organisms by free radicals, hyaluronidase, and enzyme. In short, adjustable reactive groups and HA have been extensively used in bone tissue engineering to cure cartilage defects, rheumatoid arthritis, and osteoarthritis, depending on their functionality (Jeznach et al., 2018). Gurski et al. established a 3D culture system that was biomimetic for weakly adherent bone metastatic prostate cancer cells (C4-2B) for screening of anticancer drugs *in vitro* (Gurski et al., 2009). To that purpose, complementary hydrazide (HAADH) and aldehyde (HAALD) derivatives of HA were produced and studied. C4-2B cells were encapsulated in situ by simply combining HAADH and HAALD in the presence of the cells. Unlike cells grown in 2D monolayer culture, which exhibit an unusual spread morphology, cells growing in the HA matrix developed discrete clustered structures that expanded and merged, mimicking the growth of real tumors. Anticancer drugs applied to the medium around the cell/gel composite diffused into the gel and killed the trapped cells. The HA hydrogel method was effectively utilized to investigate the efficacy of the anticancer drug such as docetaxel, camptothecin, and rapamycin, both in combination and alone, as well as specificity, dosage, and timely responses. The anti-neoplastic responses of cells in the 2D monolayer and 3D hydrogel-based HA systems were different. We suggest that results obtained from 3D HA systems are preferable to those obtained from traditional 2D monolayers because the 3D model more properly reflects the microenvironment of cancer cells' bone metastatic.

Yu et al. developed a hydrogel with curcumin (Cur) loaded chitosan (CS) nanoparticles (CCNP) encapsulated silk fibroin (SF)/HA esterified by methacrylate (HAMA) (CCNPs-SF/HAMA) for osteosarcoma therapy and bone regeneration using photocuring and ethanol treatment (Yu et al., 2021). In the CCNPs-SF/HAMA hydrogel, micro or nanofiber networks were discovered. The FTIR data showed that the alcohol vapor treatment increased the SF β-sheets, which resulted in the CCNPs-SF/HAMA hydrogel's high compressive stresses and Young's modulus. According to the water uptake investigation, SF decreased the CCNPs-SF/HAMA hydrogel water uptake slightly, whereas CCNPs increased it. Both SF and CCNPs enhanced the CCNPs-SF/HAMA hydrogel swelling ratio, as measured by the swelling kinetics. The cumulative release profile of the CCNPs-SF/HAMA hydrogel revealed that the release of Cur from the hydrogel was accelerated as the pH was reduced from 7.4 to 5.5. Additionally, the CCNPs-SF/HAMA hydrogel, as compared to CCNPs, had a longer-lasting drug release, which was desirable for long-term osteosarcoma treatment. The results of in vitro assays suggested that CCNPs-SF/HAMA hydrogel containing an equal Cur (150 g/mL) was anticancer and promoted osteoblast proliferation. These findings show that a CCNPs-SF/HAMA hydrogel with exceptional physical qualities and the ability to perform dual functions of osteosarcoma therapy and bone repair may be an attractive choice for local cancer therapy and bone regeneration.

### 4.8. Chitosan-based hydrogels

Chitosan (CS) is also a type of glycosaminoglycans (GAGs) found in cartilage tissue that interacts with signal transduction, growth factors, and adhesion cells directly or indirectly. In other words, CS is a biocompatible polymer that has been shown to aid bone regeneration (Soundarya et al., 2018). CS has the benefit of being easily changed by its glucosamine residues (e.g. carboxymethylation, acylation, and alkylation) to increase its physicochemical characteristics and biological activities. The porous nature of the scaffold facilitates cell migration and tissue regeneration, and CS’s ability to produce porous structures is another benefit. Numerous strategies have been used over the last decade to develop an injectable hydrogel capable of forming a strong scaffold in situ, including redox and photoinitiator-guided polymerization. One point worth noting is that extended exposure to initiators and irradiation will result in cytotoxicity (Fedorovich et al., 2009). As a result, it is critical to create a moderate environment for injectable hydrogel network crosslinking. Schiff’s base reaction is a moderate reaction that can result in the formation of imine bonds through amino and aldehyde groups (Balakrishnan et al., 2013). Based on Schiff’s reaction, CS can be chosen as an appropriate biomaterial to make an injectable hydrogel due to the abundance of amino groups in its structure. Schematic illustration of hydrogel-assisted bone regeneration is provided in Figure 8 (Bai et al., 2018).

Tao et al. developed a thermosensitive hydrogel derived from CS to produce a VCM-nanoparticle (NPs)/Gel local drug delivery system (Tao et al., 2020). VCM-NPs were synthesized using quaternary ammonium CS and carboxylated CS nanoparticles (VCM-NPs) to improve the encapsulation efficiency and drug loading of VCM, to prevent infection and heal fractured bones simultaneously. A rabbit osteomyelitis model was used to assess this hydrogel. The disclosed multifunctional hydrogel system for local administration demonstrated bone regeneration promoting and anti-infection properties, indicating that it has substantial promise as a scaffold for effective osteomyelitis treatment. Min et al. described the synthesis of CS-poly(dioxanone) copolymers with different amounts of poly(dioxanone) and free amino groups on the CS backbone (Min et al., 2019). The selected CS-poly(dioxanone) was combined with HA to form HA/CS-poly(dioxanone) polyelectrolyte complex NPs by an ionotropic gelation process, and this type of HA/CS-poly(dioxanone) polyelectrolyte complex NPs was used as a carrier for bone morphogenetic protein-2 (BMP-2) delivery. To achieve long-term BMP-2 release, the optimum BMP-2-encapsulated HA/CS-poly(dioxanone) NPs were embedded in CS-glycerophosphate composite solutions to form various hydrogels. The gels were determined to be injectable at room temperature and to have a thermosensitive phase transition around physiological temperatures and pH values. Additionally, they demonstrated the ability to release BMP-2 in a nearly linear manner for several weeks while effectively retaining the encapsulated BMP-2’s bioactivity. Because the currently developed gel systems are completely biocompatible and biodegradable, they have a high potential for translation to the clinic to be used in bone repair and regeneration, where persistent and controlled stimulation from active signaling molecules and stable biomechanical scaffolds for housing recruited cells are frequently required simultaneously.

### 4.9. Micro/nanogels as functional drug delivery vehicles

Because of the rapid advancement of nanotechnology in recent decades, a wide range of nanomaterials, such as liposomes, nano micelles, nanoparticles, and nano/microgels, have been used in various fields (Wu et al., 2011; Chen et al., 2016; Zhang et al., 2017, 2018). These small molecules can easily pass vessels and localize to certain tissues, and they can also be functionalized with specific molecules to enable active targeting. As a result, this system will serve as an attractive platform for biomedical applications such as bone regeneration, cartilage repair, tumor therapy, and wound healing (Ding et al., 2013; Agrawal & Agrawal, 2018; Zhu et al., 2018). Nano/microgels, in comparison to other small substances, have a controllable nanoparticle size, a cell-adhesive porous structure, good biocompatibility, and a large surface area. Additionally, their stimuli-sensitivity, adaptable functional groups, and high water content make them an excellent biomaterial for life science applications. With an increasing appreciation and application of the material’s advanced and biocompatible properties, many biomaterials employed in micro/nanogels have received considerable interest due to their distinct advantages in terms of biological activity and polymer chain modification.

Microgels have attracted substantial attention in the field of tissue engineering in recent years due to their excellent biocompatibility, cost-effectiveness, and functional diversity (Heris et al., 2016). Additionally, the ability to enwrap macromolecule proteins, polypeptides, metal/nonmetal ions, or small molecule medicines contributes to tissue regeneration. Thus, various micro-based scaffolds were utilized to facilitate cellular migration and differentiation by acting as a favorable microenvironment. Wu et al. prepared a PNIPAM-based microgel system modified by PEG and galactose ligands. Additionally, PEG was used to minimize the gel system's shrinking, and galactose ligands were combined with PNIPAM to boost the hydrogel's cell viability (Wu et al., 2014). Moreover, a human hepatocarcinoma cell line (HepG2 cells), was enwrapped in this gel scaffold, and the experimental data demonstrated the expected phenomena of increased cell viability with increasing galactose ligand content. Li et al. developed a functional gelatin-based assembly injectable microgel containing stem cells (Li et al., 2018). The crosslinker used in this system was NH-PEG, which resulted in covalent bonding between the microgel building blocks. The hBMSCs were then encapsulated in a network based on NHS-treated assembled (NHSA) microgels, which allowed them to maintain excellent cell viability while also increasing cell migration. NHSA-microgels also demonstrated a higher level of chondrogenic gene expression, suggesting that this biocompatible microgel could be used in bone regenerative therapy.

Nanogels are spherical hydrogels with a nanoscale diameter that are formed by physically or chemically interconnecting aqueous architectures. Nanogels share some characteristics with hydrogels, including excellent biodegradability and controlled mechanical properties (Elkhoury et al., 2019). Nanogels can be utilized to encapsulate bioactive substances including miRNA or protein therapeutics, preserving biological activity in vitro and resulting in a more effective therapy effect in vivo than in situ injectable hydrogels and hydrogel scaffolds (Sasaki & Akiyoshi, 2010). Nanogels, due to their nanoscale and surface area, have a higher potential in drug delivery systems than microgels. For instance, drug-loaded nanogels can be activated with targetable compounds such as folic acid, a targeted peptide, or an antibody, and then administered intravenously to tumors or osteoarthritis (Culver et al., 2016). In recent decades, stimuli-responsive nanogels, which varied their shape or other features in response to external conditions including temperature, pH, and physiochemical environment, have gained remarkable consideration in the field of bone tissue engineering, such as OA, RA, and, bone defects, as well as fracture. Temperature changes can affect the internal network of the nanogel, as well as its size and interactions with foreign particles, resulting in the controlled release of loaded biomolecules (Qureshi & Khatoon, 2019). The thermosensitive derivative N-isopropylacrylamide (NIPAm) has been extensively utilized in biomedicine (Qian & Wu, 2013). NIPAm is hydrophilic below 32 °C (its lower critical solution temperature (LCST)). When the temperature surpasses the LCST, it becomes hydrophobic due to the aggregation of NIPAm units, causing the system to shrink. Furthermore, chain changes can control the LCST of pNIPAm (Chen et al., 2013). Off-targeted, unstable, or insoluble, bioactive compounds could be encapsulated in nanogels and deposited in inflammatory regions via target recognition or an enhanced permeability and retention (EPR) effect (Wang et al., 2018).

Table 1 illustrates the various types of hydrogel used for the treatment of bone tumor as well as bone regeneration. We'll go through some of the most promising alternative hydrogel matrices that have shown great bone formation along with bone tumor therapy in several animal defect models.

**Table 1.** Examples of hydrogels used for bone tumor therapy and bone regeneration.

Biomaterial	Mode of tumor treatment	Bone Regeneration	References
Hydrogenated black TiO_2_ (H-TiO_2_) coating with biomimetic hierarchical micro/nanostructures deposited on a titanium implant	In-vitro and in-vivo, the photothermal treatment caused Sao-2 bone tumor cells necrosis.	In-vitro, BMSC adhesion, proliferation, and osteogenic differentiation were enhanced by hierarchical micro/nanotopography on an implant.	(Zhang et al., 2019)
Polydopamine and cisplatin decorating an n-HA surface loaded in chitosan/alginate hydrogels	Photothermal therapy and chemotherapy for 4T1 breast tumor-bearing mice	The bifunctional hydrogel induced bone repair in the joint bones of rabbits	(Luo et al., 2019)
Nanohydroxyapatite hybrid reduced graphene oxide (n-HA-rGO) hydrogel	The n-HA-rGO hydrogel induced photothermal therapy and killed almost all MG-63 osteosarcoma cells in vitro and in vivo	The n-HA-rGO hydrogel promoted bone regeneration with the stimulation of osteoblast mineralization and collagen deposition in a rat cranial defect model	(Saber-Samandari et al., 2019)
PECE/Collagen/n-HA hydrogel	The temperature-sensitive hydrogel significantly induced the bone regeneration in rabbits	The PECE/Collagen/n-HA hydrogel composite showed significant therapeutic efficacy and directed bone regeneration performance compared to the self-healing process.	(Fu et al., 2012)
CCNPs-SF/HAMA hydrogel	The hydrogel provided the pH triggered release and effectively killed the MG-63 cells	CNPs-SF/HAMA hydrogel promoted the osteoblast proliferation	(Yu et al., 2021)
Methacrylated gelatin/methacrylated chondroitin sulfate hydrogel hybrid gold nanorods (GNRs) and nanohydroxyapatite (n-HA),	The hydrogel induced photothermal therapy and induced apoptosis of K7M2wt cells	The hydrogel mimics the ECM, promoting mesenchymal stem cell proliferation and osteogenic differentiation.	(Liao et al., 2021)
hybrid hydrogel (UCNP-Au-Alg) via the complexation of up-conversion lanthanide-Au hybrid nanoparticles (NPs) and alginate	Photothermal induced the apoptosis of T24 cells and significantly reduced the tumor growth in mice	The elastic hybrid hydrogel facilitated the repair of the bone structure due to its supportive function	(Liu et al., 2021)
Bifunctional UV and Sr^2+^ double-crosslinked alginate (ALG)/alkylated gelatin (GelAGE) hydrogels incorporated with polydopamine (PDA) particles	Hydrogels effectively killed the MG63 osteosarcoma cells through the synergy of controlled DOX release and hyperthermia ablation.	enhance the proliferative potential of rat bone mesenchymal stem cells (rBMSCs) and also the alkaline phosphatase (ALP) activity of cells, suggesting their osteogenic promotion ability	(Chen et al., 2022)
hydrogels based on Furan-Sodium Alginate/bis-maleimide-Polyethylene Glycol/Copper doped Bioactive Glass-ceramic Microspheres (SA/PEG-CuBGM)	The hydrogel induced the photothermal therapy and effectively killed the K7M2-WT cancer cells	The SA/PEG-CuBGM composite hydrogel group showed significantly accelerated proliferation of mBMSCs and induced the most efficient bone formation	(Yang et al., 2021)
Curcumin-microsphere/IR820 coloaded hybrid methylcellulose hydrogel (Cur-MP/IR820 gel)	The Hydrogel induced hyperthermia mediated curcumin release and showed the K7M2wt osteosarcoma cells	The hydrogel indueced the bone regeneration via promoting the alkaline phosphatase expression and calcium deposition of bone mesenchymal stem cells.	(Tan et al., 2021)

## 5. Conclusion and perspective

The current review explores the recent advancements in the development of functionalized hydrogels for the treatment of bone tumors. A novel technique based on functionalized hydrogels has the potential to suppress tumor growth during the initial stages of treatment and boost bone repair during the late stages of treatment. The benefits of locally and systemically administered functionalized hydrogels have the potential to significantly enhance the survival rate of patients with bone tumors. The use of functionalized hydrogels in future clinical bone tumor treatments may offer new possibilities for increasing patient quality of life and decreasing mortality.

There are still numerous challenges that need to be resolved in actual applications in future studies. Although several investigations including in vitro or in vivo experimental studies have been undertaken to date, however, relatively few clinical trials have been conducted using a human model or human cells. To the best of our knowledge, the following are the specific reasons: (1) Hydrogels are often examined in subcutaneous models that do not accurately depict the complex microenvironment of bone diseases associated with fracture repair or major bone defect repair. (2) While the majority of the test animals are healthy young animals, the practical application of this bone tissue engineering approach is expected to occur in more mature individuals and the elderly, with certain complications. (3) In addition to model challenges, hydrogel storage is a difficult nut to crack, which adds to the complexity of using hydrogels in clinical applications. Hydrogels that swell during storage and transportation are more susceptible to disruption, which can result in medication leakage. To address this problem, dehydration following preparation is recommended; although, this method may alter the structure and properties of reswollen hydrogels. (4) Unlike natural bone tissue, most hydrogels are incapable of self-repair. This disadvantage will result in hydrogels being mechanically damaged during the implantation procedure or bone activity. (5) Numerous functional hydrogels with appropriate mechanical characteristics and high bioactivities have demonstrated beneficial therapeutic effects in vitro and in vivo. However, both the complex preparation techniques and the high cost of hydrogel scaffolds place significant constraints on their further development and use in clinical applications.

Based on the foregoing, numerous critical elements should be examined to further develop hydrogel application at the clinical stage. To begin, the rate at which hydrogels degrade should correspond to the rate at which bone defects regenerate. Second, a comprehensive and well-defined clinical criterion for tissue engineering should be developed; concurrently, quality control standards and safety evaluation for these hydrogels should be developed. Additionally, because patients' needs vary, researchers should focus on precision medicine rather than the one-size-fits-all approaches that have gradually become the dominant path of biomaterial design in bone tumor therapy and tissue engineering. Meanwhile, because bone tumor therapy and tissue engineering are complex processes, and each biomaterial has distinct advantages, researchers must select appropriate biomaterials for use as bone repair scaffolds based on the application requirements. As a result, a low-cost, cell-free, and easy-to-manufacture hydrogel system must be designed as a platform for clinical trials, eventually benefiting humans.
